# Novel species interactions: American black bears respond to Pacific herring spawn

**DOI:** 10.1186/s12898-015-0045-9

**Published:** 2015-05-26

**Authors:** Caroline Hazel Fox, Paul Charles Paquet, Thomas Edward Reimchen

**Affiliations:** Raincoast Conservation Foundation, PO Box 2429, Sidney, BC V8L 3Y3 Canada; Department of Biology, University of Victoria, PO Box 1700 STN CSC, Victoria, BC V8W 2Y2 Canada; Department of Geography, University of Victoria, PO Box 1700 STN CSC, Victoria, BC V8W 2Y2 Canada

**Keywords:** Species interactions, *Clupea pallasii*, *Ursus americanus*, Intertidal zone, Forage fish, Pacific Ocean, Spawn

## Abstract

**Background:**

In addition to the decline and extinction of the world’s species, the decline and eventual loss of species interactions is one of the major consequences of the biodiversity crisis. On the Pacific coast of North America, diminished runs of salmon (*Oncorhynchus* spp.) drive numerous marine–terrestrial interactions, many of which have been intensively studied, but marine–terrestrial interactions driven by other species remain relatively unknown. Bears (*Ursus* spp.) are major vectors of salmon into terrestrial ecosystems, but their participation in other cross-ecosystem interactions is similarly poorly described. Pacific herring (*Clupea pallasii*), a migratory forage fish in coastal marine ecosystems of the North Pacific Ocean and the dominant forage fish in British Columbia (BC), spawn in nearshore subtidal and intertidal zones. Spawn resources (eggs, milt, and spawning adults) at these events are available to coastal predators and scavengers, including terrestrial species. In this study, we investigated the interaction between American black bears (*Ursus americanus*) and Pacific herring at spawn events in Quatsino Sound, BC, Canada.

**Results:**

Using remote cameras to monitor bear activity (1,467 camera days, 29 sites, years 2010–2012) in supratidal and intertidal zones and a machine learning approach, we determined that the quantity of Pacific herring eggs in supratidal and intertidal zones was a leading predictor of black bear activity, with bears positively responding to increasing herring egg masses. Other important predictors included day of the year and Talitrid amphipod (*Traskorchestia* spp.) mass. A complementary analysis of black bear scats indicated that Pacific herring egg mass was the highest ranked predictor of egg consumption by bears. Pacific herring eggs constituted a substantial yet variable component of the early springtime diet of black bears in Quatsino Sound (frequency of occurrence 0–34%; estimated dietary content 0–63%). Other major dietary items included graminoids (grasses and sedges), Phaeophyta (brown algae), Zosteraceae (seagrasses), and Talitrid amphipods.

**Conclusion:**

This research represents the first scientific evidence of a cross-ecosystem interaction between Pacific herring and American black bears. Our findings also expand knowledge of the ecological roles of both species. Combined, evidence of anthropogenic constraints on both black bears and Pacific herring suggests that bear-herring interactions were potentially stronger and more widespread in the past.

## Background

Conservation efforts to address the consequences of the ongoing biodiversity crisis primarily focus on species extinctions and the closely related issue of species abundances. Concurrent to species extinctions however, are the loss of species interactions that may occur well before a species goes extinct [1]. Because ecosystems are built upon a web of interactions between species and their environments, the loss of species interactions can result in the simplification and degradation of ecosystems [1, 2]. Further, because ecosystems are incredibly complex and only a fraction of the world’s species has been described, the lack of information regarding species interactions, their contributions to ecosystem processes, and the pace of their extinctions is immense.

On the Pacific coast of North America, one of the more intensively studied cross-ecosystem interactions involves the migration of salmon (*Oncorhynchus* spp.). Anadromous Pacific salmon spend the majority of their lives in marine ecosystems, where they gain >99% of their mass, before re-entering complex freshwater ecosystems to spawn and die [3]. For the ecosystems that receive salmon, the ecological consequences of this spatial subsidy, defined as the movement of energy, material, and nutrients across ecosystem boundaries [4], can be profound. Known ecological consequences extend from interactions at the base of the food web (e.g., primary producers [5]), through to insects [5], small mammals [6], and songbirds [7] to apex-level predators, including Bald Eagles (*Haliaeetus leucocephalus* [8]), bears (*Ursus* spp. [9, 10]) and gray wolves (*Canis lupus* [11]). Although salmon may be transported into terrestrial areas by flooding [12], hyporheic movement [13], and the activities of terrestrial predators (gray wolves [11]; mink [6]), brown and black bears (*Ursus arctos* and *Ursus americanus*) are recognized as dominant predators and vectors of salmon [9, 14, 15].

Salmon are only seasonally available to coastal bears however, who continue to interact with the wide range of ecosystems they inhabit (e.g., bark stripping of conifers [16]). Further, many of the same traits that cause bears to be dominant predators of salmon, including their mobility, opportunism, and relative abundance in coastal landscapes, are likely related to the exploitation of other marine and intertidal organisms, including whale carcasses [17], clams, crabs, and barnacles [18, 19] by bears that access intertidal ecosystems. Despite their prominent role as vectors of salmon into terrestrial ecosystems, the participation by bears in other marine–terrestrial interactions has only been the subject of isolated speculation (e.g., [20]).

Other anadromous and nearshore spawning fishes, including Pacific herring (*Clupea pallasii*), have been suggested as a pulsed resource for marine and terrestrial organisms [21], although information relating to their cross-ecosystem influences remains largely unknown. In the coastal marine ecosystems where they occur, ranging from Baja California to Alaska and westward to Eurasia, Pacific herring can have immense ecological, cultural, and economic importance [22–24]. Highly interactive and abundant, at least historically (e.g., [24]), Pacific herring are a foundation species (sensu [1]) and in British Columbia (BC), are considered the dominant forage fish [22].

From egg to spawning adult, iteroparous Pacific herring are prey for a diversity of marine taxa, including birds, mammals, sharks, groundfish, salmon, and invertebrates (e.g., [25–30]), but it is their spawn events in particular that link herring to nearshore, intertidal, and terrestrial ecosystems. Beginning in late winter in the southern part of their range, Pacific herring aggregate and spawn in nearshore subtidal and intertidal zones, with males releasing milt (sperm) into the water column and females laying eggs directly onto subtidal and intertidal substrates. Spawn events advance northward along the Pacific coast and generally end in summer, a trend that is linked to sea surface temperatures [31]. In BC, most Pacific herring spawn from February to April. Responding to these spatiotemporally pulsed aggregations of fish and lipid-rich eggs, recently described as “egg boons” [32], are predators and scavengers, including invertebrates (e.g., [33]) and more than 25 vertebrates (reviewed by [34]). With exception to Northwestern Crows and Canada Geese (reviewed by [34]), the majority of species reported to exploit spawning Pacific herring and eggs are marine or marine-associated.

Drawing on our experiences and the scientific literature concerning interactions between terrestrial predators and salmon (e.g., [9, 11, 14]), macrophyte wrack subsidies (e.g., [35]), and subsidies associated with relatives of Pacific herring (e.g., [36–38]), we developed the broad prediction that Pacific herring interacted with terrestrial ecosystems, likely via the foraging activities of predators and/or scavengers at spawn events. In addition to soliciting anecdotal observations of terrestrial mammals consuming Pacific herring eggs from fishermen and fisheries biologists, we conducted a pilot study that documented black bears foraging extensively on Pacific herring eggs in the supratidal and intertidal zones of Quatsino Sound, BC, Canada.

With the above knowledge, this two-part study sought to identify the (1) predictors of black bear activity in supratidal and intertidal zones and (2) predictors of Pacific herring egg consumption by black bears using scats collected from intertidal, supratidal, and adjacent terrestrial areas. Black bear use of supratidal and intertidal zones was measured using remote cameras positioned on beaches in Quatsino Sound (spring 2010–2012), a number of which received varying amounts of Pacific herring eggs. Using the machine learning (ML) algorithm random forests (RF; [39]), predictors of black bear activity included major intertidal diet items (Pacific herring egg and amphipod mass), gray wolf activity (measure of risk), day of the year, year, and location. Because major supratidal and intertidal zone dietary items were either previously unknown or only infrequently reported in the scientific literature, an independent dataset based on scat was particularly relevant. Our second objective was to assess the predictors of Pacific herring egg consumption by black bears, which was measured using estimated dietary content in bear scats. This second objective provides corroborative evidence for camera-derived results but also assesses the dietary importance of Pacific herring to black bears. Potential predictors of Pacific herring egg estimated dietary content in black bear scats were assessed using RF and included herring egg and amphipod masses, day of the year, year, and location.

## Methods

### Study area

Quatsino Sound, located off northwest Vancouver Island, BC, Canada was chosen as the study area due to the presence of a small but reliable Pacific herring spawn and somewhat intact coastal ecosystems. Pacific herring spawn annually in Quatsino Sound in March or April. In recent years, an estimated ~1,000 metric tons of Pacific herring spawn in Quatsino Sound nearshore subtidal and intertidal zones [40, 41], which represents a small meta-population relative to other herring meta-populations in BC. During the study period, there was relatively low human presence on beaches and adjacent terrestrial areas; low use by researchers associated with this study constituted the majority of human presence.

**Figure 1:**
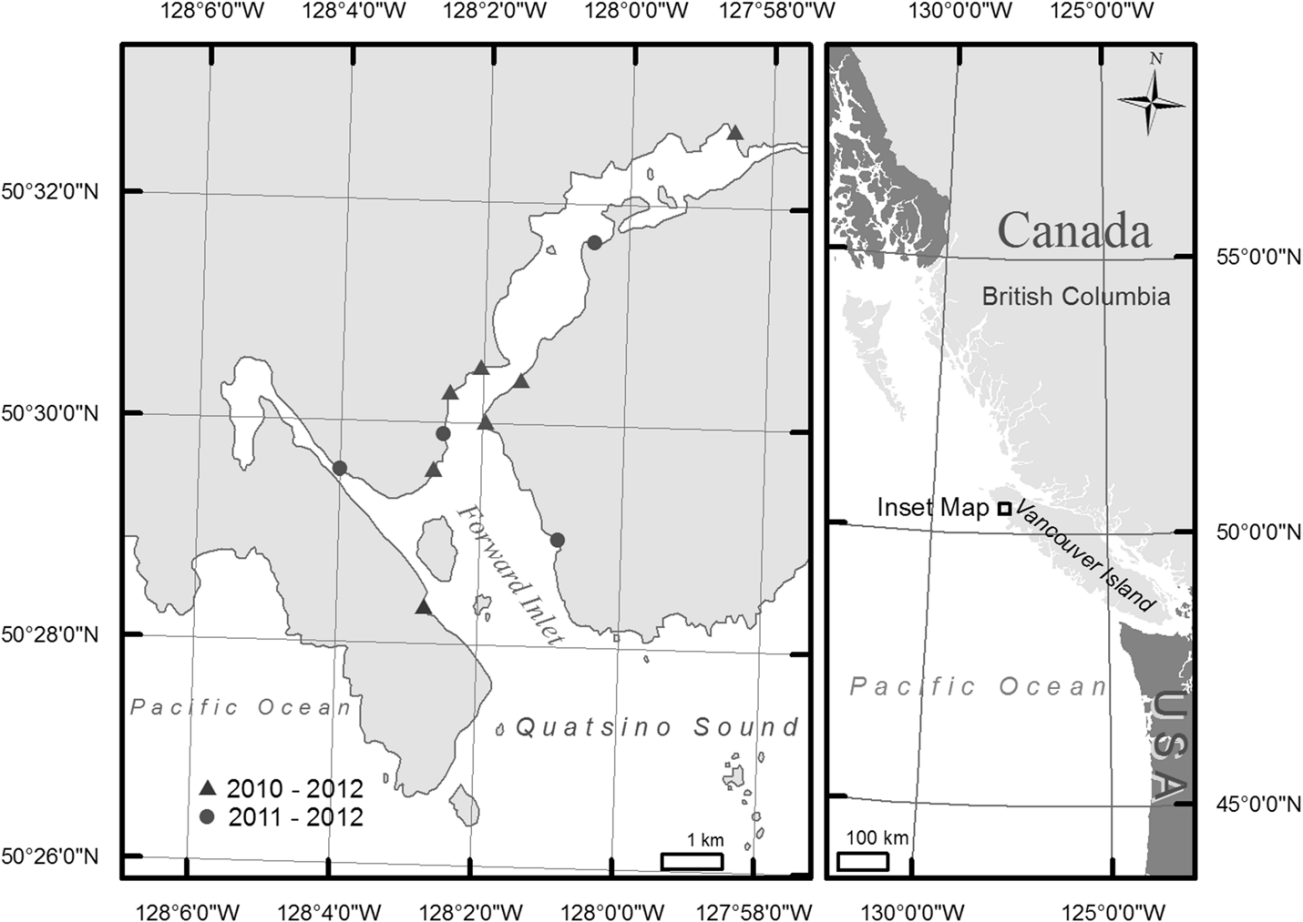
Location of study sites in Quatsino Sound, British Columbia, Canada (2010–2012). Figure generated using ArcGIS v.10 (ESRI, Redlands, CA).

The study period was March to early May (2010, March 2–May 3, day of the year 61–123; 2011, March 7–May 6, day of the year 66–126; 2012, March 7–April 27, day of the year 67–118). Study site locations consisted of seven beaches in 2010 and 11 beaches in 2011 and 2012 (Figure 1). Sites were selected on the basis of several factors: cumulatively, beaches were (1) a mix of spawn/spawn-free, (2) representative of the regional supratidal and intertidal habitat accessible by bears, (3) not within sight of commercial roe-on-kelp operations (to reduce disturbance), and (4) accessible by boat or foot. Substrates on all beaches were mixed. With the exception of one estuarine-type site with a broad, grass-covered supratidal zone, beaches had narrow (<4 m) supratidal zones filled with logs and wrack with old growth or mature second growth forest in the adjacent terrestrial area. Exposure on beaches varied, ranging from exposed, open-ocean swell to sheltered, where waves were unlikely to exceed 0.5 m. The tidal cycle in the region is mixed semidiurnal. In Quatsino Sound, the maximum tidal height is approximately 4.3 m.

### Beach surveys

Although Pacific herring spawn directly on subtidal and intertidal substrates, only intertidal and supratidal zones were surveyed. In a related study of Quatsino Sound black bears, >100 h of observations indicated that bears spend the majority of their time foraging in supratidal and intertidal zones and consume eggs laid directly on intertidal substrates but also eggs deposited in supratidal and intertidal zones by wind, waves and tidal action (CHF, unpublished data). Further, we note that while there are numerous methods for enumerating Pacific herring eggs, most modern methods are intended for fisheries management purposes (e.g., [42]) and/or rely on SCUBA (e.g., [43, 44]), the latter of which is inappropriate for supratidal zones. To meet objectives for this and related studies (e.g., [33, 45]), we used stratified random quadrat and sediment core sampling surveys of supratidal and intertidal zones for two major black bear food sources, Pacific herring eggs and *Traskorchestia* spp. amphipods.

Evidence of spawn on all beaches was first assessed by visual inspection of the water for milt, aggregations of Pacific herring and/or attached eggs on nearshore subtidal and intertidal substrates in multiple periods before and during the herring spawn period. On beaches with evidence of spawn, egg mass, a measure of quantity, was estimated along a 100 m portion of beach using 25 randomly distributed 0.25 m^2^ quadrats during the spawn period (prior to egg hatch). Along five random perpendicular transects, three quadrats were randomly assigned to tidal heights in the low (1–2 m), mid (2–3 m) and high (3–4 m), in addition to two quadrats in the fresh (within the 3–4 m zone) and the older supratidal wrack lines (above 4.3 m, lowest low water). Within each quadrat, an estimate of egg number was generated using visual estimates of percent cover of ‘loose’ and ‘attached’ eggs, where eggs are affixed to macrophytes (wrack and attached) or other substrates (e.g., eggs laid on boulders). Loose egg percent cover was estimated and number of egg layers (egg depth) randomly counted at five locations and averaged. Attached egg cover was estimated, egg layers counted on five random pieces of macrophyte vegetation and layers of macrophytes with eggs counted at five random locations.

To generate a cumulative estimate of number of eggs within each quadrat, percent cover of loose eggs and attached eggs were converted to number of eggs using an egg radius (r = 0.74 mm) from Alderdice and Hourston [46], which was similar to our egg radius measurement made on site in Quatsino Sound (r = 0.83 mm, n = 20). Values from Alderdice and Hourston [46] were used as these were judged conservative relative to our samples, which were saturated with freshwater. The total number of loose eggs (number of loose eggs × average number of egg layers) was added to total number of attached eggs (number of attached eggs × average number of egg layers × average number of macrophyte layers). Egg samples collected in 2012 (10 eggs per sample, n = 5) were dried at 60°C for 48 h and weighed, with mean mass per egg (0.27 mg) used to calculate egg mass for a 1 m wide section of beach perpendicular to the coastline (supratidal to the low intertidal zone). Egg abundance or mass could be generated for any length of beach; we used egg mass and a 1 m wide section of beach for simplicity of comparison.

Eggs are lost from spawning grounds via consumption by predators and scavengers, removal by wind and waves, degradation, and other mechanisms. Egg loss rates at Pacific herring spawns in BC have been previously measured (e.g., [47, 48]) but because egg loss rates vary, site or region-specific egg loss rates have been recommended [48]. Egg surveys were repeated at beach study sites during the pre, hatch, and post egg hatch periods and 12 daily egg loss rates were estimated (2011, 4 beaches, 12 surveys, 8 egg loss rates estimated; 2012, 2 beaches, 6 surveys, 4 egg loss rates estimated). Because no significant differences in egg loss rates were detected in the pre and post hatch periods (ANOVA; F_1,10_ = 4.04, p = 0.07), an average daily egg loss rate was calculated (6.9% ± 1.1 SE). For the first 5 days of the spawn, we conservatively estimated a 20% daily linear increase in egg mass across all beaches to the estimated spawn maxima on day five. This 20% estimated increase over 5 days was used for two reasons: (1) the gradual addition of eggs into the supratidal and intertidal was observed during active spawning (~5 days in Quatsino Sound) and (2) tidal, wind and wave action washes eggs into supratidal and intertidal zones, with elevated and often irregular egg loading in the early days of the spawn (CHF, pers. obs.).

Unlike Pacific herring eggs, *Traskorchestia* spp. amphipods (max. body length = 2 cm) are generally restricted to the upper intertidal and supratidal zones and commonly occur in high densities under wrack (CHF, unpublished data). Immediately adjacent to quadrats in the high (3–4 m), fresh, and degraded wrack lines, sediment cores (diameter = 10.6 cm) were used to collect amphipods in beach sediment down to 10 cm depth (n = 15 per beach). Beaches were sampled for amphipods once, with a preference for sampling later in the study period, when amphipods were active and accessible by black bears. Samples were frozen at −20°C until processing. Samples were sieved through a 0.75 mm mesh under running water, 30% of sample was retained, spread over a large surface and amphipods manually removed. Amphipods were dried at 60°C for 48 h and weighed. Similar to herring eggs, amphipod mass was calculated for a 1 m wide section of beach perpendicular to the shoreline. Mass, a measure of quantity, was used due to the high variability of beach widths and the restricted vertical habitat of amphipods. Amphipod mass on individual beaches was assumed to be constant over the study period for two reasons: (1) the majority of mass consisted of adults from the overwintering population and (2) juveniles had not yet recruited to the adult-sized population and were excluded by the 0.75 mm mesh size.

### Remote cameras

The use of remote cameras (i.e., camera-trapping) is an established non-invasive approach for monitoring wildlife, including the activity of brown and black bears at salmon streams (e.g., [49, 50]). Two camera models (Reconyx RM30 and HC500, Wisconsin, USA; n = 7–11 per year; 1 camera per beach) equipped with infrared motion sensors and an infrared flash to allow for image capture during darkness were used with identical settings (24 h active mode, three rapid-succession images captured per trigger, high trigger sensitivity). For the duration of the study period, cameras were affixed to trees adjacent to beach study sites at heights (~1–1.5 m) and positioned such that large mammals in supratidal and intertidal zones would be detected. After positioning, all cameras were checked for detection of large mammals using the ‘walk-test’ function with human subjects in supratidal and high intertidal zones. Cameras were checked every 1–2 weeks; during camera checks memory cards were collected, batteries replaced and cameras re-positioned as necessary. All camera photos were date and time stamped.

To assess camera model bias, the two models were first tested using human subjects. At distances approximating the supratidal (1 m), high (5 m) and mid (10 m) intertidal, both camera models displayed 100% successful detection of human subjects. At distances of 15 m and greater, RM30 routinely failed to detect human subjects at distances that approximated the low intertidal. However, in practice, neither camera model captured images of any large mammals in the low intertidal. Further, two cameras (one of each model) were set out on a beach at 5 m horizontal distance for 41 days and a comparison of camera detections of black bears revealed no significant difference (Wilcoxon signed-rank test; Z = −1.31, p = 1.91). As such, the influence of camera model bias in the low intertidal was judged negligible.

Observations of black bears during a pilot study in 2009 found that bears spent an average of 35 ± 6 SE minutes in supratidal and intertidal zones (CHF, unpublished data). Apart from females with cubs, the majority of black bears observed in supratidal and intertidal zones were alone; the occurrence of multiple bears on the same beach was only observed twice and these animals were >150 m apart (CHF, pers. obs.). Subsequently, black bear activity was based on a period of 30 min; multiple bear detections at less than 30 min were recorded as a single event and bears detected at intervals greater than 30 min scored as independent events. Black bear activity was then summed for a cumulative daily count. Black bears with cubs were counted as one animal. Days for individual cameras that experienced malfunction or significant repositioning by black bears were excluded.

Cameras also detected gray wolves and their often elevated use of the supratidal and intertidal warranted their examination as a potential predictor of black bear activity. Individual wolves were not identified due to the poorer quality of camera images during darkness. Wolf activity was generated on the same 30-min basis as bears, without regard to the number of individuals detected, and then summed per day. For comparison, we also recorded the activity other large mammals (black-tailed deer, *Odocoileus hemionus columbianus* and cougar, *Puma concolor*) on a 30-min basis.

### Scat surveys

In 2010, black bear scat surveys were conducted at all sites throughout March and into early May; this protocol was modified in 2011 and 2012 for surveys to begin after the first bear was detected. Cameras marked the centre of the scat survey, with 250 m of supratidal and intertidal zones on either side of the cameras searched. At each beach, five wildlife trails that ran roughly perpendicular to the beach were also searched (100 m length each). In total, scat surveys at each site consisted of 500 m beach and 500 m wildlife trails. Sites were searched 2–3 times each year, with the timing of searches intended to capture a ~14 day window of fresh scats per location.

The upper 80% of scats aged less than an estimated 14 days were collected and frozen until processing. Scat analysis generally followed the methods outlined by Dahle et al. [51] and Persson et al. [52]. Scats were washed in a 0.75 mm sieve, mixed by hand and five, six ml volumes (measured by water displacement) subsampled per scat. Subsamples were dispersed over a 1 cm^2^ grid with ~15 ml of water. Dietary components were identified, their volume estimated and subsequently averaged. Previous research has confirmed that visual estimates of percent volume correlates well with exact measurements [53]. Scat items were summarized by year using frequency of occurrence (FO) and percent faecal volume (FV):

$${\text{FO}}_{\text{X}} = \frac{{{\text{number}}\;{\text{of}}\;{\text{scats}}\;{\text{containing}}\;{\text{item}}\;{\text{X}}}}{{{\text{total}}\;{\text{number}}\;{\text{of}}\;{\text{scats}}}}{ \times } 1 0 0 {\text{\% }}$$

$${\text{FV}}_{\text{X}} = \frac{{{\text{mean}}\;{\text{volume}}\;{\text{of}}\;{\text{food}}\;{\text{item}}\;{\text{X}}}}{{{\text{total}}\;{\text{faecal}}\;{\text{volume}}}}{ \times } 1 0 0 {\text{\% }}$$

Dietary items differ in their digestibility, with highly digestible items underestimated and poorly digestible items often overestimated [52]. To address this, correction factors that relate faecal volume with original dietary intake are commonly applied [54]. Annual faecal volumes were multiplied with a number of diet-specific correction factors (CF_1_) to generate estimates of original diet as estimated dietary content in percent dry mass. Estimated dietary content was then multiplied by a second correction factor (CF_2_) that relates dry matter to energy (kJ/g dry weight), expressed as estimated dietary energy content.

Estimated dietary content and estimated dietary energy content were estimated based on the conservative assumption that macroalgae and seagrass consumption by black bears confers energy. Although direct observation of black bears suggests that consumption of marine macrophytes (seagrass and macroalgae) is incidental to the consumption of Pacific herring eggs and amphipods and that most marine macrophytes found in scat appear to undergo minimal digestion (CHF, pers. obs.), energetic benefit could not be ruled out. Polar bears are also known to consume macroalgae, but leading explanations include vitamin and mineral uptake rather than energetic benefit [55]. Overall, we know little of algal digestibility by bears.

Correction factors (CF_1_) used to estimate estimated dietary content from faecal volume were obtained from a diet study of brown bears (graminoids = 0.24, forbs = 0.26, arthropods = 1.1, and whole trout = 40.8; [54]). Pritchard and Robbins [56] found no significant differences in digestive or metabolic efficiencies between brown and black bears and other studies have applied brown bear correction factors to black bears (e.g., [57]). Correction factors for Pacific herring eggs, macroalgae and seagrasses are not known; we used the values for whole trout (40.8) and graminoids (0.24) respectively, and interpret results with caution. In particular, we interpret the application of the trout correction factor (CF_1_) for Pacific herring eggs as conservative for several reasons. First, unlike trout, eggs have no hard parts to consistently survive digestion and are primarily composed of protein, lipids, and water. Second, black bear digestibility of protein in similar items is very high [56]. Third, several scats consisted almost exclusively of seagrasses and macrophytes with zero eggs and zero to trace amphipods and yet black bears were never observed to target marine macrophytes unless eggs or amphipods were present (CHF, pers. obs.).

To convert estimated dietary content to estimated dietary energy content, we used a second round of correction factors (CF_2_, kJ/g), with herring eggs = 18.7 [28], graminoids = 6.3, forbs = 8.4, arthropods = 11.3, except ants = 17.7 [51] and talitrid amphipods = 10.8 [58]. Correction factors for macroalgae = 13.14 [58] and seagrasses = 16.8 [59] were also applied, the latter of which was based on *Zostera marina*. Excluded from all estimated dietary content and estimated dietary energy content calculations were molluscs, bryozoa, black bear hair, feathers, trees/shrubs, bryophyta, gravel, and garbage, as these were judged to contribute negligible energy to black bears. Molluscs represented small faecal volumes and were limited to worn shell fragments. Bryozoa, feathers, and bryophytes were similarly evaluated, as they constituted very small faecal volumes and were readily found in wrack lines. Tree and shrub remains constituted somewhat larger faecal volumes, but the majority of items were identified as small woody debris and conifer needles, both of which are abundant in wrack lines. Black bear hair represented trace faecal volumes and was attributed to grooming by bears rather than consumption of conspecifics. Lastly, garbage was limited to small worn fragments of hard plastic and Styrofoam^®^, which was indicative of incidental ingestion in supratidal and intertidal zones. During the study period, black bears in the area had limited access to human garbage.

### Statistical analysis

The machine learning (ML) algorithm random forests (RF; [39]) was used to identify predictors for two response variables, black bear activity (summed on a daily basis per beach) and Pacific herring egg estimated dietary content in scats (mean herring egg estimated dietary content per scat collection day for each study site location). Regression RF is an ensemble of decision trees that combines bagging, here the independent construction of successive trees, and the random selection of predictor variables [39]. For our purposes, this approach provides a pseudo R^2^, a rank importance of predictor variables, and a visual assessment (partial dependency plots) of the marginal effect of a predictor on the response variable. Two separate regression RFs with 700 trees and two variables tried at each split were performed using the package randomForest version 4.6-7 [60] in the software program R [61]. Variable importance was determined using two measures. The first, percent increase in mean standard error (MSE), records the prediction error on the permuted out-of-bag data for each predictor variable. The difference between the predictor variable and the permuted predictor variable prediction error is averaged over all trees and normalized by the standard deviation of differences. The second measure of variable importance uses the total decrease in node impurities derived from splitting on the variable, averaged over all trees and measured by residual sum of squares. For black bear activity, predictors included day of the year, year, location, activity of gray wolves (daily sum of detections), and mass (kg) of amphipods and Pacific herring eggs within a 1 m wide section of beach perpendicular to the shoreline. For Pacific herring egg estimated dietary content in scats (per beach study site location and scat collection date), predictors included amphipod mass, mean egg mass for the 14 days before and including the scat collection date, day of the year, year and location. SPSS v.21 (IBM, Armonk, NY) and ArcGIS v.10 (ESRI, Redlands, CA) were also used to visualize data and produce a map of the study area, respectively.

## Results

### Bear activity in supratidal and intertidal zones

**Figure 2:**
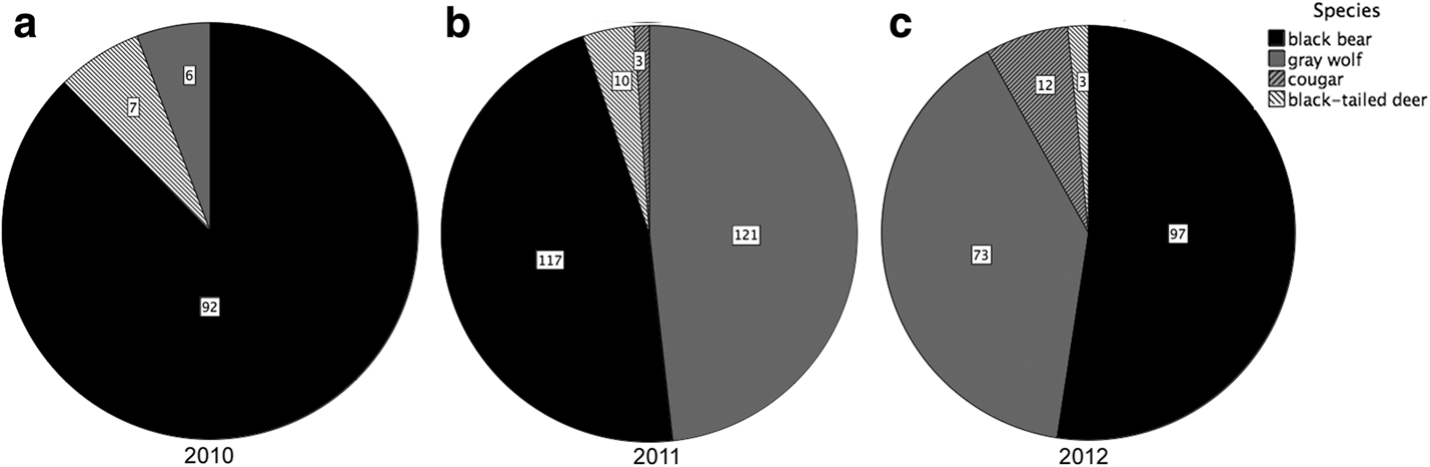
Summary of camera-trapped large mammals in supratidal and intertidal zones in **a** 2010, **b** 2011 and **c** 2012. Cameras (2010, n = 7, 422 camera days; 2011, n = 11, 545 camera days; 2012, n = 11, 500 camera days) were located on beaches in Quatsino Sound, British Columbia, Canada. Counts for large mammals reflect independent camera detections on a 30-min basis (see “Methods”).

In 2010, black bears were the most frequently camera-trapped large mammal in supratidal and intertidal zones with only relatively small numbers of black-tailed deer and gray wolves detected (Figure 2a). Black bears still made up a large proportion of mammals detected in the supratidal and intertidal in 2011 and 2012, but in these years, a wolf pack moved into the area (Figure 2b, c). In 2010, the majority of wolves captured on camera were lone individuals but in the following 2 years, multiple individuals were commonly photographed in the same frame. With few exceptions, black bears could not be individually identified using camera images, but from direct observations of individual bears over the course of the study, we conservatively estimate that a minimum of 15 individuals were present each year (CHF, pers. obs.). All black bears appeared to access supratidal and intertidal zones, including large adult males, which typically were the first to emerge, and later, smaller bears, subadults, and females with cubs. We note that one beach appeared to be dominated by a large adult male bear (CHF, pers. obs. and camera images), with evidence of winter den and springtime ‘day den’ use ([62], CHF, pers. obs.) in the adjacent forest.

**Figure 3:**
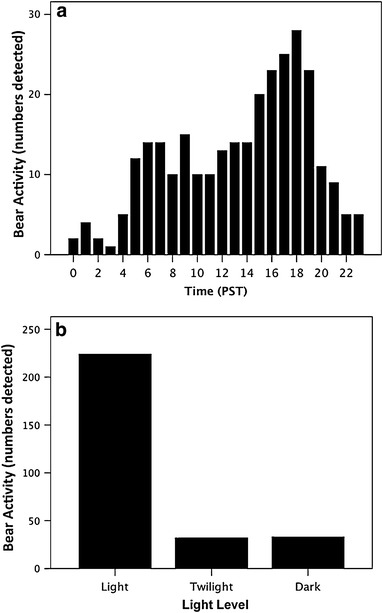
Cumulative black bear activity in supratidal and intertidal zones by **a** hour and **b** light level. Black bear activity is a measure of independent detections (30 min intervals) of bears by remote cameras positioned on beaches in Quatsino Sound, British Columbia (2010–2012). Twilight is inclusive to both nautical and civil twilight.

**Figure 4:**
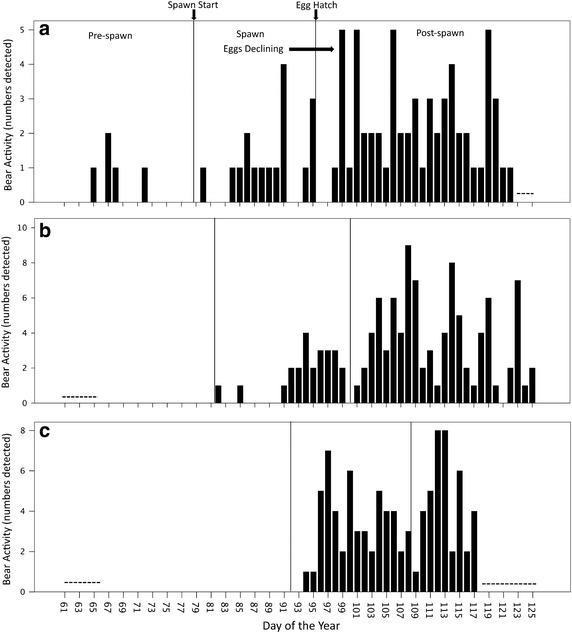
Cumulative daily activity of black bears and the spawning period of Pacific herring in **a** 2010, **b** 2011, and **c** 2012. Black bear daily activity is the daily sum of independent detections (30 min intervals) of bears by remote cameras positioned on beaches in Quatsino Sound, British Columbia (2010, n = 7 cameras; 2011, n = 11; 2012, n = 11). The study period was March to early May (2010, March 2–May 3, day of the year 61–123; 2011, March 7–May 6, day of the year 66–126; 2012, March 7–April 27, day of the year 67–118). *Dashed lines* indicate no data (cameras not operational).

Black bears were largely diurnal, with highest supratidal and intertidal activity in the late afternoon and early evening (Figure 3a, b). Timing of black bear emergence onto beaches was variable, with bears first detected on camera in early March (day of the year = 65, March 6) in 2010 but not until late March (day of the year = 82, March 23) in 2011 and April in 2012 (day of the year = 94, April 3; Figure 4a–c). Timing of black bear emergence onto beaches coincided somewhat with the onset of the Pacific herring spawn, which began March 20 (day of the year = 79) in 2010, March 22 (day of the year = 81) in 2011 and April 1 (day of the year = 92) in 2012 (Figure 4a–c).

Major supratidal and intertidal dietary items available to black bears varied annually. In 2010, Pacific herring eggs were found on five of seven beaches, averaging 0.05 kg (d.w.) ± 0.03 (SE) per 1 m wide section of beach perpendicular to the shoreline. In 2011 and 2012, Pacific herring spawned on six and eight beaches, respectively (2011, n = 6 of 11 beaches; 2012, n = 8 of 11 beaches), averaging 1.18 kg (d.w.) ± 0.77 in 2011 and 1.10 kg (d.w.) ± 0.61 (SE) in 2012. Amphipods were found on most beaches (26 of 29), averaging 0.14 kg (d.w.) ± 0.08 in 2010, 0.06 kg (d.w.) ± 0.02 in 2011 and 0.03 kg (d.w.) ± 0.01 (SE) in 2012.

**Figure 5:**
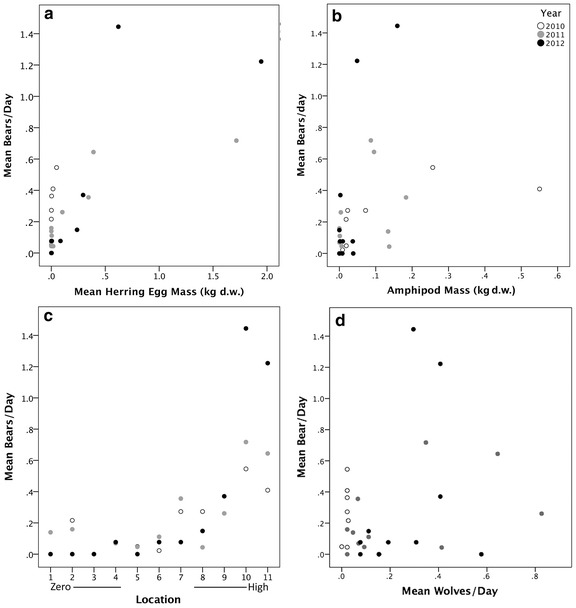
Relationship between mean black bear activity per day and potential predictors during Pacific herring spawn and post-spawn periods. Mean black bear activity for each beach study site and year with **a** Pacific herring egg mass (kg d.w.), **b** amphipod (*Traskorchestia* spp.) mass (kg d.w.), **c** location, ranked from lowest to highest mean Pacific herring egg mass, and **d** mean number of gray wolves per day. Mean black bear activity per day represents an average of the cumulative daily number independent bear detections (30 min intervals) by remote cameras positioned on beaches in Quatsino Sound, British Columbia (2010, n = 7 cameras; 2011, n = 11; 2012, n = 11).

RF was used to quantitatively assess predictors of daily black bear activity at beaches but we also used cumulative bear counts over the study period with leading predictors to provide a visual assessment of these relationships. Black bear activity was greater at beaches with higher Pacific herring egg masses and although more variable, bears also tended to be more frequent at beaches with higher amphipod masses (Figure 5a, b). The relationship between black bear activity and location, ranked from zero to higher average Pacific herring egg mass, was positive (Figure 5c). Although antagonistic interactions between gray wolves and black bears were opportunistically observed (CHF, pers. obs.), no relationship between the two species was detected, although we note that this relationship may be apparent on different spatiotemporal scales (Figure 5d).

**Figure 6:**
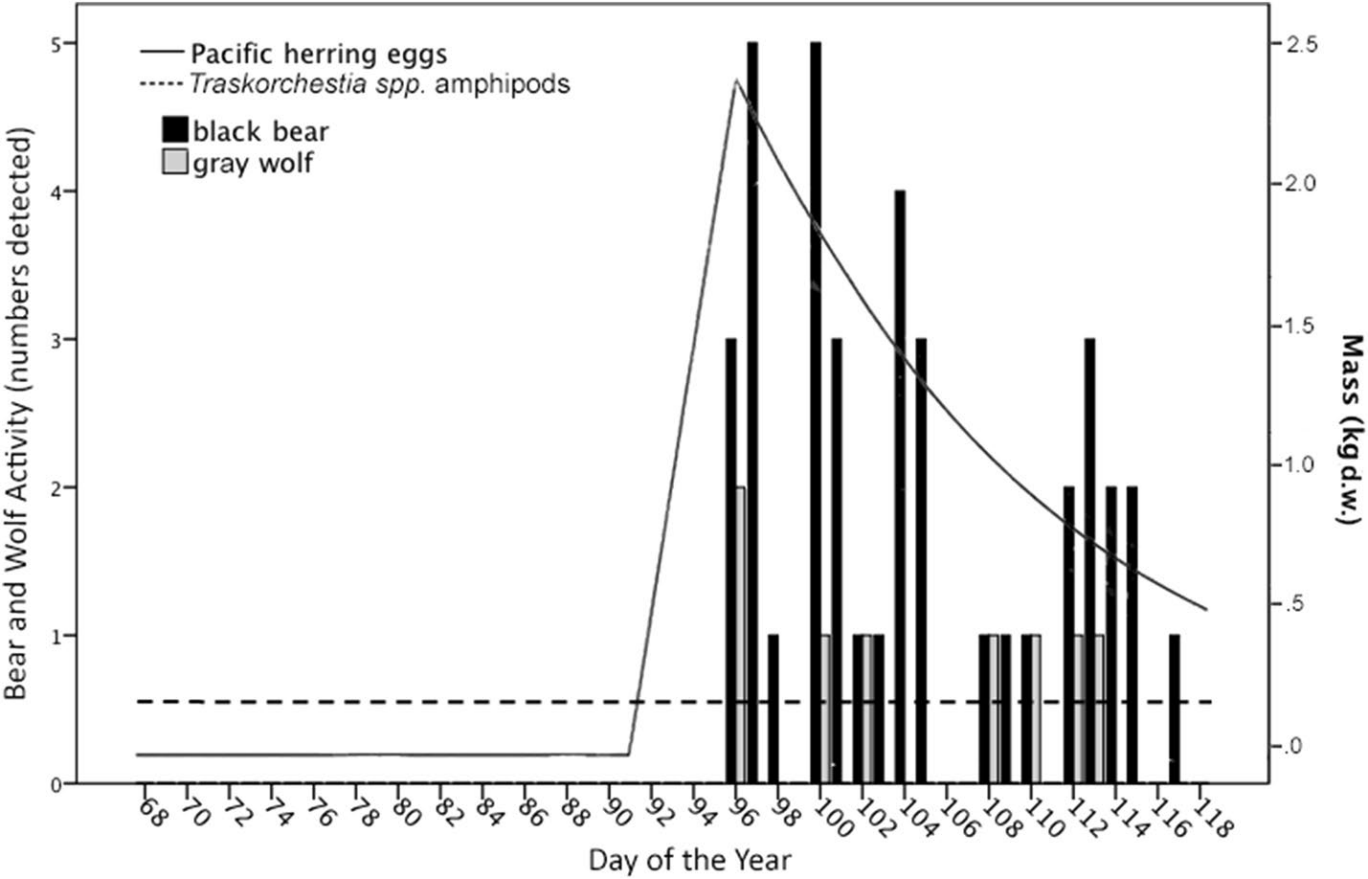
Black bear and gray wolf activity per day at a representative beach with Pacific herring egg and *Traskorchestia* spp. amphipod masses (kg d.w.). Black bear and gray wolf activity are cumulative daily independent detections (30 min intervals) of bears and wolves by a remote camera positioned on a single beach study site in Quatsino Sound, British Columbia (2012). Amphipod mass is an assumed constant at each beach (see “Methods”).

**Figure 7:**
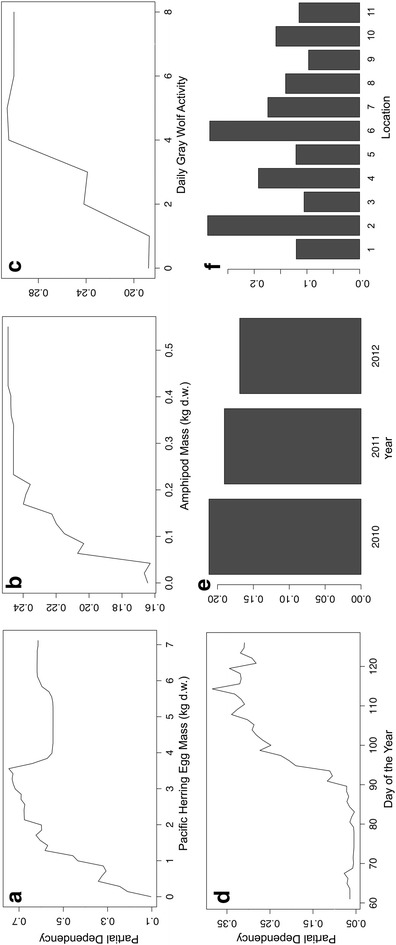
Random forest partial dependencies for black bear activity with predictor variables. Partial dependencies of black bear activity (mean daily count per beach and year) with **a** Pacific herring egg mass (kg d.w.), **b** amphipod (*Traskorchestia* spp.) mass (kg d.w.), **c** daily gray wolf activity, **d** day of the year, **e** year and **f** beach location in Quatsino Sound, British Columbia, Canada.

For black bear activity in the intertidal, RF models were not constructed using cumulative sums; instead, bear activity and associated predictors were analyzed on a daily basis per beach. Data for one spawn beach in 2012, where both black bears and gray wolves were relatively abundant, are plotted to illustrate model structure (Figure 6). Using percent increase MSE to rank variable importance, the top two ranked predictors of black bear activity were day of the year (33.4) and Pacific herring egg mass (26.1), followed by similar, moderate-importance predictors year (14.7), amphipod mass (16.8), and location (16.7) with gray wolf activity as the lowest predictor (3.8). Variable rank importance using increase in node purity was similar, with Pacific herring egg mass (146.7) and day of the year (121.7) as highly-ranked predictors, followed by location (47.3), amphipod mass (36.4), gray wolf activity (25.6) with year as the lowest-ranked predictor (17.5). Averaged pseudo R^2^ was moderate (30.7%). Partial dependencies of predictor variable influence on black bear activity illustrate that elevated Pacific herring egg and amphipod masses, the latter part of the study period, elevated gray wolf activity, and certain beach study locations had greater influences on bear activity (Figure 7a–f).

### Scats

A total of 163 bear scats were analyzed for dietary content (2010, n = 35, 7 sites; 2011, n = 81, 11 sites; 2012, n = 47, 11 sites). Percent faecal occurrence was judged the least informative, with high faecal occurrences for multiple diet items, including macroalgae, arthropods, grasses and sedges (graminoids), and gravel (Table 1). In terms of faecal volumes, major dietary components included brown algae (2.9–38.6%), amphipods (5.4–24.5%), seagrasses (9.8–39.5%), and graminoids (5.9–56.9%; Table 1). Pacific herring eggs were absent from scats in 2010 and constituted trace amounts in 2011 and 1.1% faecal volumes in 2012 (Table 1).

**Table 1:** Black bear scat summary results for 2010, 2011, and 2012

Taxa/group	Common name	FO			FV		
		2010	2011	2012	2010	2011	2012
Phaeophyta	Brown algae	*48.6*	*53.1*	*95.7*	*2.9*	*12.6*	*38.6*
*Macrocystis integrifolia*	Giant kelp	2.9	32.1	72.3	0.6	8.6	25.0
*Egregia menziesii*	Feather boa kelp	–	–	46.8	–	–	5.8
*Fucus* spp.	Rockweed	17.1	18.5	46.8	tr.	0.6	1.8
Other Laminariales	Kelps	45.7	39.5	66.0	1.9	3.4	6.1
Chlorophyta	Green algae	*2.9*	*21.0*	*10.6*	*tr.*	*0.7*	*tr.*
Rhodophyta	Red algae	–	*21.0*	*42.6*	*–*	*0.5*	*0.6*
Arthropoda	Invertebrates	*74.3*	*39.5*	*44.7*	*25.1*	*7.8*	*5.5*
Insecta	Insects	–	1.23	–	–	tr.	–
Coleoptera	Beetles	31.4	7.4	19.1	tr.	tr.	tr.
Diptera	Flies	–	2.5	2.1	–	tr.	tr.
Formicidae	Ants	2.9	–	–	tr.	–	–
Decapoda	Crabs	–	1.2	–	–	tr.	–
*Hemigrapsus* spp.	Shore crabs	8.6	2.5	–	tr.	tr.	–
Talitridae	Amphipods	65.7	35.8	51.1	24.5	7.8	5.4
Cirripedia	Barnacles	2.9	–	–	tr.	–	–
*Lepas* spp.	Pelagic gooseneck barnacles	2.9	–	–	tr.	–	–
Mollusca	Molluscs	*5.7*	*8.6*	*8.5*	*tr.*	*tr.*	*tr.*
Other Mollusca		5.7	4.9	6.4	tr.	tr.	tr.
Bivalvia	Clams and oysters	–	1.2	2.1	–	tr.	tr.
Gastropoda	Snails	–	3.7	–	–	tr.	–
Bryozoa	Marine bryozoans	–	1.2	–	–	tr.	–
Chordata	Animals	*5.7*	*21.0*	*34.0*	*tr.*	*tr.*	*1.1*
*Clupea pallasii*	Pacific herring eggs	–	12.3	34.0	–	tr.	1.1
*Ursus americanus*	Black bear hair	5.7	4.9	2.1	tr.	tr.	tr.
Aves	Feathers	–	3.7	–	–	tr.	–
Streptophyta	Land plants	*100*	*100*	*100*	*70.1*	*75.0*	*51.4*
Zosteraceae	Seagrasses	48.5	58.0	97.9	9.8	18.7	39.5
Graminoids	Grasses and sedges	94.3	63.0	25.5	56.9	50.0	5.9
Trees and shrubs	Needles and wood	77.1	70.3	97.9	1.4	5.4	4.9
Bryophyta	Mosses	2.9	11.1	2.1	tr.	tr.	tr.
Forbs		2.9	3.7	–	2.0	1.0	1.1
Gravel		*40.0*	*48.1*	*70.2*	*1.8*	*2.7*	*2.7*
Garbage		*8.6*	*4.9*	*4.3*	*tr.*	*tr.*	*tr.*

Taxa/group	Common name	EDC			EDEC		
		2010	2011	2012	2010	2011	2012
Phaeophyta	Brown algae	*1.3*	*8.6*	*13.2*	*1.8*	*9.1*	*10.3*
*Macrocystis integrifolia*	Giant kelp	tr.	5.3	8.4	tr.	5.7	6.6
*Egregia menziesii*	Feather boa kelp	–	–	1.9	–	–	1.5
*Fucus* spp.	Rockweed	–	tr.	0.6	–	tr.	tr.
Other Laminariales	Kelps	1.0	2.1	2.0	1.4	2.2	1.6
Chlorophyta	Green algae	*–*	*tr.*	*–*	*–*	*0.5*	*–*
Rhodophyta	Red algae	*–*	*tr.*	*tr.*	*–*	*tr.*	*tr.*
Arthropoda	Invertebrates	*61.7*	*22.2*	*8.4*	*68.4*	*19.5*	*5.4*
Insecta	Insects	–	tr.	–	–	tr.	–
Coleoptera	Beetles	tr.	tr.	tr.	0.6	tr.	tr.
Diptera	Flies	–	tr.	tr.	–	tr.	tr.
Formicidae	Ants	tr.	–	–	tr.	–	–
Decapoda	Crabs	–	tr.	–	–	tr.	–
*Hemigrapsus* spp.	Shore crabs	0.7	tr.	–	0.9	tr.	–
Talitridae	Amphipods	60.2	22.1	8.3	66.6	19.3	5.4
Cirripedia	Barnacles	tr.	–	–	tr.	–	–
*Lepas* spp.	Pelagic gooseneck barnacles	tr.	–	–	tr.	–	–
Mollusca	Molluscs						
Other Mollusca							
Bivalvia	Clams and oysters						
Gastropoda	Snails						
Bryozoa	Marine bryozoans						
Chordata	Animals	*–*	*25.8*	*62.7*	*–*	*39.2*	*70.0*
*Clupea pallasii*	Pacific herring eggs	–	25.8	62.7	–	39.2	70.0
*Ursus americanus*	Black bear hair						
Aves	Feathers						
Streptophyta	Land plants	*36.9*	*43.2*	*15.6*	*28.8*	*31.7*	*14.0*
Zosteraceae	Seagrasses	5.3	11.6	13.3	9.1	15.8	13.3
Graminoids	Grasses and sedges	30.5	31.0	2.0	19.7	15.9	0.7
Trees and shrubs	Needles and wood						
Bryophyta	Mosses						
Forbs		1.2	0.7	tr.	1.0	tr.	tr.
Gravel							
Garbage							

Reported values represent percent frequency of occurrence (FO), percent faecal volume (FV), percent estimated dietary content (EDC) and percent estimated dietary energy content (EDEC) of dietary items in black bear scats collected in Quatsino Sound, British Columbia in 2010 (n = 35, 7 sites), 2011 (n = 81, 11 sites) and 2012 (n = 47, 11 sites). Items constituting <0.5% are indicated with trace (tr.). Italics text indicates cumulative values for the taxonomic group shown. EDC and EDEC values were not calculated for molluscs, bryozoans, black bear hair, feathers, needles and wood, mosses, gravel or garbage (see “Methods”).

Using estimated dietary content, amphipods (8.3–60.2%), Pacific herring eggs (0.0–62.7%), and graminoids (2.0–31.0%) were identified as major dietary items over the 3 year study (Table 1). In 2010, amphipods constituted the majority of the estimated dietary content (60.2%), with 30.5% represented by graminoids. In 2011, contributions were more balanced, with 22.1% from amphipods, 25.8% from Pacific herring eggs, and 31.0% from graminoids but in 2012, herring eggs represented 62.7% of the diet, with only minor contributions from brown algae, amphipods, and graminoids (Table 1). Estimated dietary energy content calculations resulted in similar patterns, with dominant contributions from amphipods (66.6%) and graminoids (19.7%) in 2010 and lesser amounts in 2011 (amphipods = 19.3%, graminoids = 15.9%), with most energy derived from Pacific herring eggs (39.2%; Table 1). In 2012, Pacific herring eggs dominated the diet of bears, in terms of estimated dietary energy content (70.0%), with minor contributions from brown algae, amphipods and graminoids (Table 1).

**Figure 8:**
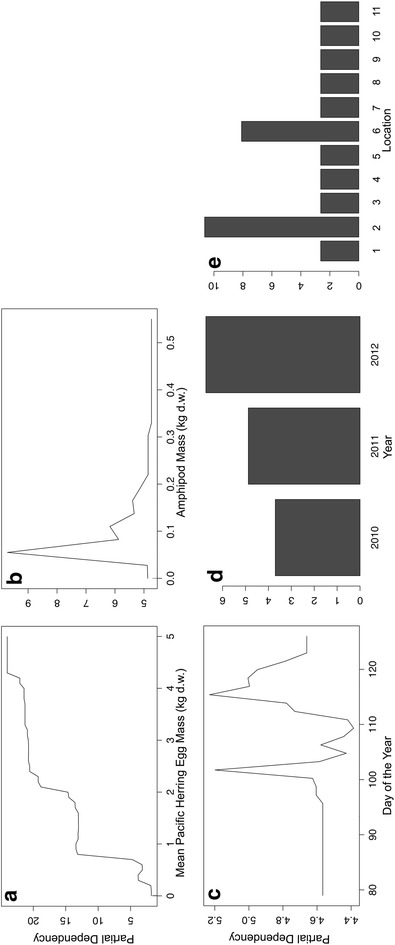
Random forest partial dependencies of Pacific herring egg estimated dietary content (EDC) with predictor variables. Partial dependencies of Pacific herring egg estimated dietary content (EDC) with **a** 14-day mean Pacific herring egg mass (kg d.w.), **b** amphipod (*Traskorchestia* spp.) mass (kg d.w.), **c** day of the year, **d** year and **e** beach location in Quatsino Sound, British Columbia, Canada. Pacific herring egg EDC was obtained from an analysis of black bear scats (n = 163).

Using the estimated dietary content of Pacific herring eggs in scats as the response variable and ranking variable importance using percent increase MSE in the RF analysis, the top ranked predictor was averaged Pacific herring egg mass (16.6) followed by moderate predictors location (10.4), amphipod mass (10.3), and year (8.2) with day of the year as the lowest-ranked predictor (−4.6). Variable rank importance using increase in node purity was similar, with averaged Pacific herring egg mass (6779.6) ranked as the top predictor, followed by moderate predictors location (2171.7), amphipod mass (1972.6), and year (1082.5) with day of the year as the lowest-ranked predictor (856.7). Pseudo R^2^ was moderate (40.9%). Partial dependencies of predictor variable influences on Pacific herring egg estimated dietary content in scats illustrate that elevated herring egg mass, lower amphipod mass, later days, certain beach study site locations and the latter 2 years of the study period had greater influences (Figure 8a–e).

## Discussion

Following emergence from dens, black bears were frequent users of Quatsino Sound supratidal and intertidal zones. Of all the large mammals detected in 2010, black bears were the most common. In 2011 and 2012, gray wolves and black bears were both frequent visitors to supratidal and intertidal zones. As in other parts of their range [63], black bears were primarily diurnal and exhibited limited activity during twilight and darkness, in contrast to primarily nocturnal activity by black bears foraging on Pacific salmon [64].

Using RF, we determined that day of the year and Pacific herring egg mass were highly ranked predictors of black bear activity. The high relative importance of day of the year was expected, given that the emergence of black bears from dens occurred during the study period, with bears being rare for most of March. Black bears responded to Pacific herring spawn events, as indicated by the high importance ranking of herring egg mass, with greater activity on beaches with greater quantities of herring eggs. In relative terms, amphipod mass was ranked as moderate predictor of black bear activity, indicating that bears were also responding to their quantity.

Paired with the examination of supratidal and intertidal bear activity, the collection of black bear scats provided complementary evidence of the reliance by bears on Pacific herring eggs and amphipods. In 2010, the majority of energy was obtained from amphipods, which were relatively abundant on beaches. In turn, generally low Pacific herring egg masses coincided with an absence of eggs in scats collected in 2010. Although we acknowledge uncertainty over the use of the trout correction factor [54] as a proxy for Pacific herring eggs, evidence that black bear digestion of protein in similar items is high [56] supports our opinion that our dietary approach was conservative and likely underestimated egg consumption. Still, estimated dietary content and estimated dietary energetic content values are interpreted with caution. Overall, scat analysis indicated that black bears were more reliant on Pacific herring eggs in the last 2 years of the study; these greater energetic contributions coincided with greater egg quantities in comparison to 2010 and likely reflects the flexible exploitation of localized abundances of a high quality resource by bears. Providing corroborative evidence of the importance of Pacific herring eggs to the springtime diets of black bears, mean Pacific herring egg mass was the strongest predictor of egg estimated dietary content in bear scats with moderate predictors including location, year and amphipod mass. The lower ranking of amphipod relative to Pacific herring egg mass further suggests that bears are responding to the amount of eggs.

When combined with previous reports, our understanding of the importance of amphipods and Pacific herring eggs in influencing the activity and diet of black bears is only marginally improved, mainly because both prey items are relatively unknown. Pacific herrings eggs have not been previously described in the scientific literature as a resource for bears, although black bears have been observed consuming eggs elsewhere in BC (e.g., C. Fort and M. Hessing-Lewis, pers. comm.). Consumption of Talitrid amphipods is somewhat more established, with reports for black bears in BC (*Traskorchestia traskiana* [20, 65]) and brown bears in Siberia (Gammarid amphipods [66]) and Alaska (*T. traskiana* [17]). Van Daele et al. [17] likened brown bear exploitation of *T. traskiana* amphipods as the ecological equivalent of myrmecophagy (termite and ant consumption), which is important for some brown and black bear populations (e.g., [67, 68]).

Our analysis considers Pacific herring eggs and amphipods to be independent and relies on mass, a measure of quantity, with no reference to quality. On Quatsino Sound beaches that experience Pacific herring spawn however, the quality of *Traskorchestia* spp. amphipods can be significantly influenced. Based on carbon and nitrogen stable isotopes, an estimated 7–38% of amphipod diets on beaches experiencing spawn in 2011 were derived from Pacific herring [33]. In addition, amphipods exposed to Pacific herring spawn contained greater amounts of biologically valuable docosahexaenoic and eicosapentaenoic fatty acids, which were also attributed to herring [33]. With black bear emergence onto beaches somewhat coincident with the timing of the spawn in Quatsino, subsequent consumption of amphipods by bears on spawn beaches represents an indirect trophic link between bears and Pacific herring. Otherwise, major sources of production for amphipods were determined to be macrophyte wrack, including giant kelp (*Macrocystis integrifolia*) and surfgrass (*Phyllospadix serrulatus*), which represents a spatial subsidy to amphipods [33] and indirectly, the black bears that consume them.

Neither American black bears nor Pacific herring are at risk of extinction, but for reasons that relate to anthropogenic influences on both marine and terrestrial ecosystems, these two species are likely less interactive now than in the past. Across North America, black bear distribution has declined by 39% [69]. In locations where Pacific herring still spawn, black bears may have limited to no access due to anthropogenic disturbance (e.g., [70]), habitat degradation/loss, and localized reductions of black bear populations. In terms of herring, quantitative estimates of Pacific herring populations before industrial harvesting began (late 1800s [71]) do not exist, but a recent archaeological study of ancient human settlements in BC, Washington and Alaska indicates that herring were more abundant and exhibited greater distributional consistency prior to industrial harvesting [24]. Following a BC-wide crash in the 1960s and subsequent recovery, Pacific herring populations declined again in the 1990s and 2000s for reasons that remain poorly understood (e.g., [22]). In recent years, BC commercial fisheries have concentrated efforts on the two largest Pacific herring meta-populations. Following lengthy commercial fisheries closures, the remaining three major meta-populations in BC recently experienced modest increases and commercial fisheries were subsequently reopened (e.g., [72]), but the sustainability of these harvests remains controversial (e.g., [73]).

Because Pacific herring abundance has also been shown to influence the temporal delivery of spawn to nearshore and intertidal ecosystems, reduced herring abundances may result in reduced temporal contact between herring and intertidal and terrestrial ecosystems. Pacific herring can spawn in waves, with between-spawn periods lasting up to 26 days [74] or possibly more (I. McKechnie, pers. comm.). Further, spawning may be more drawn out in years with larger Pacific herring populations [74]. Given that eggs from a small spawn event in Quatsino Sound were observed to persist in supratidal and intertidal zones for at least 5 weeks, including the post-hatch stage [45], evidence of greater historical Pacific herring abundances [24] raises the prospect that waves of spawn events could have provided resources to coastal consumers for several months.

Combined, evidence that pre-industrial Pacific herring populations were likely more abundant, exhibited greater distributional consistency [24] and that spawn events may have occurred in ‘waves’ or in a more protracted pattern, suggests that interactions between spawning herring and terrestrial ecosystems, including bears, may have more widespread than at present. Relative to both deep time (e.g., [24]) and short term baselines (e.g., [22, 40, 41]), and regardless of the drivers (e.g., industrial harvest, climate change, etc.), reduced Pacific herring abundance equates directly with decreased spawning adults, eggs and milt with consequences that include the subsequent reduction of herring spawn resources for coastal consumers, including black bears. Using the poor Quatsino Sound spawn in 2010 as evidence, low supratidal and intertidal zone egg quantities resulted in a weakened interaction with black bears, who instead relied on amphipods and graminoids as major dietary items.

Forage fish, including Pacific herring, have fundamental importance to marine socio-ecological systems, including supporting many upper trophic level marine populations and the human fisheries that directly and indirectly target them. The cross-ecosystem consequences of forage fish, however, are rarely known. Further, despite their status as the dominant forage fish in BC, Pacific herring are managed without ecosystem-based considerations, in part due to a lack of information [41]. Given the relatively widespread occurrence of black bears in western North America coincident to the spawning areas of Pacific herring (Baja to Alaska), our study considerably broadens the scope of conservation concern, which now extends beyond marine ecosystems to include interactions with black bears and terrestrial ecosystems in general. Further, with anthropogenic restriction of both participants, the remaining areas where Pacific herring, black bears, and potentially other terrestrial species still interact warrant additional study and consideration.

## Conclusions

At Pacific herring spawning grounds in Quatsino Sound, BC, Canada, American black bears, following emergence from winter dens, demonstrated increased activity at Pacific herring spawns with greater amounts of eggs in supratidal and intertidal zones. Pacific herring egg mass was also a lead predictor of black bear consumption of eggs, which provides corroborative evidence that bears respond positively to increasing amounts of herring eggs. Combined, this is the first scientific evidence of a cross-ecosystem interaction between Pacific herring and American black bears, two relatively conspicuous species that play substantive roles in coastal ecosystems but which have not been previously linked. Potentially stronger and more widespread historically, these novel bear-herring interactions highlight the paucity of knowledge regarding species and ecosystem interactions and in turn, the lack of information regarding their potential decline.

## Abbreviations

BC: British Columbia
ML: machine learning
MSE: mean standard error
RF: random forests.
